# Early versus late surgical treatment of pelvic and acetabular fractures a five-year follow-up of 419 patients

**DOI:** 10.1186/s12891-023-06977-8

**Published:** 2023-10-27

**Authors:** Anders Enocson, Natalie Lundin

**Affiliations:** 1 Department of Molecular Medicine and Surgery, Karolinska Institute, Stockholm, Sweden; 2 Department of Trauma, Acute Surgery and Orthopaedics, Karolinska University Hospital, 17164 Stockholm, Sweden

**Keywords:** Pelvic fracture, Acetabular fracture, Trauma, Surgical treatment

## Abstract

**Background:**

Surgical treatment of pelvic and acetabular fractures is an advanced intervention with a high risk of subsequent complications. These patients are often polytrauma patients with multiple injuries in several organ systems. The optimal timing for the definitive surgery of these fractures has been debated. The primary aim of this study was to investigate the influence of timing of definitive surgery on the rate of unplanned reoperations. Secondary aims included its influence on the occurrence of adverse events and mortality.

**Methods:**

All patients from 18 years with a surgically treated pelvic or acetabular fracture operated at the Karolinska University Hospital in Sweden during 2010 to 2019 were identified and included. Data was collected through review of medical records and radiographs. Logistic regression analysis was performed to evaluate factors associated with unplanned reoperations and other adverse events.

**Results:**

A total of 419 patients with definitive surgical treatment within 1 month of a pelvic (n = 191, 46%) or an acetabular (n = 228, 54%) fracture were included. The majority of the patients were males (n = 298, 71%) and the mean (SD, range) age was 53.3 (19, 18–94) years. A total of 194 (46%) patients had their surgery within 72 h (early surgery group), and 225 (54%) later than 72 h (late surgery group) after the injury. 95 patients (23%) had an unplanned reoperation. There was no difference in the reoperation rate between early (n = 44, 23%) and late (n = 51, 23%) surgery group (p = 1.0). A total of 148 patients (35%) had any kind of adverse event not requiring reoperation. The rate was 32% (n = 62) in the early, and 38% (n = 86) in the late surgery group (p = 0.2). When adjusting for relevant factors in regression analyses, no associations were found that increased the risk for reoperation or other adverse events. The 30-day mortality was 2.1% (n = 4) for the early and 2.2% (n = 5) for the late surgery group (p = 1.0). The 1-year mortality was 4.1% (n = 8) for the early and 7.6% (n = 17) for the late surgery group (p = 0.2).

**Conclusions:**

Early (within 72 h) definitive surgery of patients with pelvic or acetabular fractures seems safe with regard to risk for reoperation, other adverse events and mortality.

## Background

Surgical treatment of pelvic and acetabular fractures requires advanced multidisciplinary care and are typically centralized to regional trauma centers. Complications after surgery are commonly seen and ranges from infections, thromboembolic events to unplanned reoperations and death [1–3]. Patients with pelvic and acetabular fractures requiring surgery are often polytrauma patients with multiple injuries in several organ systems. It has recently been consented that early fixation of major fractures is crucial in polytrauma patients [4]. A stable fixation of the pelvic ring and restoration of the joint congruency of the acetabulum are important for mobilization of these patients, thereby avoiding complications associated with prolonged immobilization. However, as the surgery of pelvic and acetabular fractures is highly complex, the optimal timing has been debated. It has been recommended by many authors to delay definitive surgery with reference to a risk for major bleeding and complications [5–11]. Whereas other have advocated that early definitive surgery is safe [12–15]. In addition, a diversity exists in the literature on the definition of early versus late timing, and 8 h up to 1 week has been used as a cut-off [16–18].

The primary aim of this study was to investigate the influence of timing of definitive surgery on the rate of unplanned reoperations after pelvic and acetabular fracture surgery. Secondary aims included its influence on the occurrence of adverse events and mortality.

## Patients and methods

All adult patients (≥18) years with a pelvic or an acetabular fracture that were surgically treated within 1 month (31 days) after the injury at the Karolinska University Hospital in Stockholm, Sweden from 2010 to 2019 were included in this retrospective cohort study. Non-Swedish residents were excluded due to uncertain follow-up. Patients were selected through the local surgical database and all medical records were manually reviewed. Follow-up time was from injury date until December 31 2020, or death.

Collected demographic variables included patient age, gender and ASA-class. Injury variables collected were date and time of injury, injury mechanism, concomitant injuries, vital parameters upon arrival (systolic blood pressure, pulse rate, Glasgow Coma Scale, hemoglobin level). The trauma mechanism (high-energy or low-energy) was estimated using the 2021 US national guideline for the field triage of injured patients [19]. Pelvic fractures were classified according to Young-Burgess as anteroposterior compression (APC), lateral compression (LC), vertical shear (VS) [20], or as combined (pelvic and acetabular). Acetabular fractures were classified according to Judet and Letournel as one of five simple or five complex types [21]. Fractures were classified preoperatively by the surgeon performing the operation and confirmed by both authors reviewing the preoperative computer tomography (CT) scans. Treatment variables were date and starting time of definitive surgery and estimated intraoperative blood loss (mL) as calculated by the theatre anesthesiology staff. Follow-op variables included any unplanned reoperation including causes and types, any adverse event not requiring surgical treatment (nerve injury, pneumonia, pulmonary embolism, deep venous thrombosis, urinary tract infection, sepsis, kidney failure, superficial wound infection). In addition, length of stay at hospital and mortality at 30 days and 1 year were recorded.

## Statistical methods

Numerical data was presented as mean (SD, range) or median (IQR), unless otherwise stated. Categorical data was presented as frequency with percent distribution. Nominal variables were tested with the Fisher’s exact test. The Mann-Whitney U-test was used for comparison of scale variables in independent groups. All tests were two-sided. Logistic regression analysis was performed to evaluate factors associated with unplanned reoperations and other adverse events. Age (< 60 or ≥ 60 years), gender (male or female), trauma mechanism (high-energy or low-energy), fracture type (pelvic or acetabular) and time to definitive surgery (0–72 or > 72 h). First, crude association for each variable was tested in univariable models. Second, a multivariable model was used to study the adjusted associations. The associations were presented as odds ratios (ORs) with 95% confidence intervals (CIs). The results were considered significant at p < 0.05. The statistical software used was IBM SPSS Statistics, Version 25 for Windows (SPSS Inc., Chicago, Illinois).

## Results

### Epidemiology and injury characteristics

A total of 419 patients with a surgically treated pelvic (n = 191, 46%) or acetabular (n = 228, 54%) fracture were included. The majority of the patients were males (n = 298, 71%) and the mean age was 53 (19, 18–94) years. The median (IQR, range) time from injury to definitive surgery for all patients was 96 (96, 4-744) hours. A total of 194 (46%) patients had their surgery within 72 h (early surgery group) (median 48 (26) hours), and 225 (54%) later than 72 h (late surgery group) (median 144 (84) hours) after the injury. The median follow-up time was 1876 (1840) days (5.1 years).

A high fall (> approx. 2 m) was the most common (n = 99, 24%) mechanism of injury, followed by a simple fall (n = 85, 20%) and a car related accident (n = 70, 17%). Other vehicle related mechanisms (pedestrian hit by car, cyclist hit by car, snowmobile accident etc.) accounted for 12% (n = 50) of the injuries, motorcycle accidents for 9.3% (n = 39), horse accidents for 5.0% (n = 21) and other mechanisms for 13% (n = 55). Most patients (n = 314, 75%) had a high-energy trauma mechanism. 7.2% of the patients (n = 29) exhibited signs of circulatory shock upon arrival with systolic blood pressure < 90mmHg. Associated injuries were common; 33% (n = 140) had a concomitant chest injury, 20% (n = 85) a head or neck injury, 18% (n = 74) a major lower limb injury, 15% (n = 64) a major upper limb injury, 14% (n = 57) an abdominal injury and 13% (n = 53) a major spine injury. Epidemiology and injury characteristics in relation to timing of surgery are presented in Table 1.

**Table 1:** Epidemiology, injury characteristics and vital parameters on arrival in relation to early (within 72 h) or late (after 72 h) definitive surgery

Variable	Early surgery patients (n = 194)	Late surgery patients (n = 225)	P-value
Age; Mean (SD, range)	50 (19, 19–90)	56 (19, 18–94)	**< 0.001**
Age ≥60; n= (%)	64 (33)	108 (48)	**0.002**
Gender Female; n= (%)	57 (29)	64 (28)	0.9
ASA 3–4; n (%)	61 (31)	77 (34)	0.6
Injury mechanism; n= (%)			
Simple fall	23 (12)	62 (28)	
High fall	55 (28)	44 (20)	
Car related	30 (16)	40 (18)	
Motorcycle related	25 (13)	14 (6.2)	
Other vehicle related	25 (13)	25 (11)	
Horse related	13 (6.7)	8 (3.6)	
Other	23 (12)	32 (14)	NA
High-energy trauma mechanism; n= (%)	162 (84)	152 (68)	**< 0.001**
GCS; Median (IQR)^1^	15 (1)	15 (0)	0.4
GCS < 9; n= (%)	17 (8.9)	30 (13)	0.2
SBP (mmHg); Median (IQR)^1^	123 (33)	125 (30)	0.7
Shock; n= (%)	9 (4.8)	20 (9.2)	0.1
Pulse rate; Median (IQR)^1^	83 (25)	82 (23)	0.3
Hb (g/L); Median (IQR)^1^	127 (26)	120 (27)	**< 0.001**
Pelvic fracture; n= (%)	97 (50)	94 (42)	0.1
Head or neck injury; n= (%)	40 (21)	45 (20)	0.9
Chest injury; n= (%)	70 (36)	70 (31)	0.3
Abdominal injury; n= (%)	23 (12)	34 (15)	0.4
Major spine injury; n= (%)	32 (17)	21 (9.3)	**0.04**
Major upper limb injury; n= (%)	31 (16)	33 (15)	0.8
Major lower limb injury; n= (%)	37 (19)	37 (16)	0.5

SD = standard deviation, IQR = interquartile range, ASA = American Society of Anesthesiologists, GCS = Glasgow Coma Scale, SBP = systolic blood pressure, Hb = hemoglobin, NA = not applicable

^1^ Number of missing cases in each group: GCS n = 4, 1, SBP n = 7, 8, Pulse rate n = 7, 11, Hb n = 7, 11

### Fracture types and treatment

Pelvic fractures were classified as: APC (n = 48/191, 25%), vertical shear (n = 45/191, 24%), lateral compression (n = 42/191, 22%), combined pelvic and acetabular (n = 36/191, 19%), other (n = 7/191, 3.7%) or unable to classify (n = 13/191, 6.8%). Acetabular fractures were classified as: anterior column with posterior hemi transverse (n = 50/228, 22%), associated both column (n = 49/228, 22%), posterior wall (n = 44/228, 19%), anterior column (n = 35/228, 15%), transverse with posterior wall (n = 18/228, 7.9%), anterior wall (n = 9/228, 3.9%), posterior wall with posterior column (n = 9/228, 3.9%), t type (n = 6/228, 2.6%), transverse (n = 5/228, 2.2%), posterior column (n = 2/228, 0.9%) or unable to classify (n = 1/228, 0.4%). Detailed data on fracture types in relation to timing of surgery is presented in Table 2.

**Table 2:** Fracture types in relation to timing of surgery

Fracture type	All patients n= (%)	Early surgery patients n= (%)	Late surgery patients n= (%)
**Pelvic fractures**	**191 (46)**	**97 (50)**	**94 (42)**
APC-1	2 (0.5)	2 (1.0)	0
APC-2	31 (7.4)	18 (9.3)	13 (5.8)
APC-3	15 (3.6)	5 (2.6)	10 (4.4)
LC-1	3 (0.7)	3 (1.5)	0
LC-2	24 (5.7)	12 (6.2)	12 (5.3)
LC-3	15 (3.6)	7 (3.6)	8 (3.6)
VS	45 (11)	23 (12)	22 (9.8)
Combined	36 (8.6)	17 (8.8)	19 (8.4)
Other	7 (1.6)	3 (1.5)	4 (1.8)
Unable to classify	13 (3.1)	7 (3.6)	6 (2.7)
**Acetabular fractures**	**228 (54)**	**97 (50)**	**131 (58)**
Posterior wall	44 (11)	26 (13)	18 (8.0)
Posterior column	2 (0.5)	0	2 (0.9)
Anterior wall	9 (2.1)	4 (2.1)	5 (2.2)
Anterior column	35 (8.4)	9 (4.6)	26 (12)
Transverse	5 (1.2)	1 (0.5)	4 (1.8)
Transverse and posterior wall	18 (4.3)	8 (4.1)	10 (4.4)
Posterior column and wall	9 (2.1)	2 (1.0)	7 (3.1)
T type	6 (1.4)	3 (1.5)	3 (1.3)
Anterior column and posterior hemitransverse	50 (12)	17 (8.8)	33 (15)
Associated both column	49 (12)	26 (13)	23 (10)
Unable to classify	1 (0.2)	1 (0.5)	0

APC = anteroposterior compression, LC = lateral compression, VS = vertical shear, Combined = combined pelvic and acetabular fracture

Treatment methods used for pelvic fractures were: plating (n = 142/191, 74%), SI (sacroiliac)-screw (n = 100/191, 52%), separate screw/s (n = 38/191, 20%) and spinopelvic (n = 20/191, 11%). Treatment methods for acetabular fractures were; plating (n = 191/228, 84%), separate screw/s (n = 84/228, 37%) and total hip arthroplasty (THA) (n = 54/228, 24%). Of the 54 patients treated with THA; 51% (n = 28/54) had a reinforcement cage, 39% (n = 21/54 had a reinforcement cage and plating, 5.5% (n = 3/54) had THA only and 3.7% (n = 2/54) had THA and plating.

### Reoperations

A total of 95 patients (23%) had an unplanned reoperation. There was no difference in the reoperation rate between early (n = 44, 23%) and late (n = 51, 23%) surgery groups (p = 1.0). The most common reasons for reoperations were: infection (n = 27, 6.4%), osteoarthritis (n = 17, 4.1%) and mal-placed implant (n = 16, 3.8%). Reoperations in relation to timing of surgery is presented in Table 3. The median (IQR, range) time to the first reoperation was 38 (305, 0-1675) days. 34 patients had multiple reoperations ranging from 2 to 10 additional surgeries. The main reason for these multiple reoperations was infection that required repeated debridement’s (n = 25 patients). In order to evaluate factors contributing to an increased risk for reoperation logistic regression analysis was performed. Age, gender, trauma mechanism, fracture type and time to definitive surgery were tested. Female gender was associated with an increased risk for reoperation in univariable analysis (OR 1.7, 95% CI 1.1–2.8, p = 0.03) and in multivariable analysis (OR 1.7, 95% CI 1.0-2.8, p = 0.03). None of the other tested variables were associated with an increased risk for reoperation in uni- or multivariable analyses.

**Table 3:** Unplanned reoperations in relation to timing of surgery

Indication for reoperation	All patients (n = 419) n= (%)	Early surgery patients (n = 194) n= (%)	Late surgery patients (n = 225) n= (%)
Infection	27 (6.4)	14 (7.2)	13 (5.8)
Osteoarthritis	17 (4.1)	7 (3.6)	10 (4.4)
Malplaced implant	16 (3.8)	8 (4.1)	8 (3.6)
Disturbing implant	14 (3.3)	8 (4.1)	6 (2.7)
Failure of osteosynthesis	8 (1.9)	5 (2.6)	3 (1.3)
Dislocation of hip arthroplasty	6 (1.4)	0	6 (2.7)
Heterotopic ossification	3 (0.7)	0	3 (1.3)
Persisting fragment in the hip joint	3 (0.7)	2 (1.0)	1 (0.4)
Bleeding	1 (0.2)	0	1 (0.4)
**All**	**95 (23)**	**44 (23)**	**51 (23)**

### Adverse events, intraoperative bleeding, hospital length of stay and mortality

A total of 148 patients (35%) had any kind of adverse event not requiring reoperation. The rate was 32% (n = 62) in the early and 38% (n = 86) in the late surgery groups (p = 0.2). The most common adverse event was nerve injury (n = 63, 15%), followed by pneumonia (n = 44, 11%) and pulmonary embolism (n = 30, 7.2%). Adverse events in relation to timing of surgery is presented in Table 4. In order to evaluate factors contributing to an increased risk for adverse events logistic regression analysis was performed. Age, gender, trauma mechanism, fracture type and time to definitive surgery were tested. None of the tested variables were associated with an increased risk for adverse event in uni- or multivariable analyses.

**Table 4:** Adverse events not requiring reoperation in relation to timing of surgery

Adverse event	All patients (n = 419) n= (%)	Early surgery patients (n = 194) n= (%)	Late surgery patients (n = 225) n= (%)
Nerve injury	63 (15)	26 (13)	37 (16)
Pneumonia	44 (11)	21 (11)	23 (10)
Pulmonary embolism	30 (7.2)	13 (6.7)	17 (7.6)
Urinary tract infection	20 (4.8)	8 (4.1)	12 (5.3)
Deep venous thrombosis	16 (3.8)	7 (3.6)	9 (4.0)
Sepsis	11 (2.6)	4 (2.1)	7 (3.1)
Superficial wound infection	8 (1.9)	5 (2.6)	3 (1.3)
Kidney failure	7 (1.7)	2 (1.0)	5 (2.2)
**All** ^**1**^	**199 (47)**	**86 (44)**	**113 (50)**

^1^ Patients could have > 1 adverse event

48 patients (12%) had another major operation (open reduction with internal fixation) performed at the same time as the pelvic/acetabular surgery. The median estimated intraoperative bleeding for patients who did not have another major simultaneous operation was 720 (850) mL for the early surgery group (n = 159) and 575 (700) mL for the late surgery group (n = 196) (p = 0.01). In total, 35% (n = 147) of all patients needed intensive care; 76 (39%) in the early and 71 (32%) in the late surgery group (p = 0.1). The median total hospital length of stay was 10 (13) days for all patients; 10 (10) in the early and 9 (15) in the late surgery group (p = 0.6). The 30-day mortality was 2.1% (n = 4) for the early and 2.2% (n = 5) for the late surgery group (p = 1.0). The 1-year mortality was 4.1% (n = 8) for the early and 7.6% (n = 17) for the late surgery group (p = 0.2).

## Discussion

The main finding of this study was that no major differences in outcomes could be found when comparing early (within 72 h) and late (after 72 h) definitive surgery in patients with pelvic or acetabular fractures. In fact, the only significant difference found was that estimated intraoperative bleeding was slightly larger (difference in median values: 145 mL) in the early surgery group.

The optimal timing of surgery for these patients has been a topic for debate since long. In a literature review by Katsoulis and Giannoudis they conclude that a major problem is that the terminology of “early/late fixation” has been used highly inconsistently [7]. In general, there seem to have been a trend to operate earlier during later years. Although Latenser et al. as early as 1991 reported reduced hospital stay, long-term disability, decreased blood loss and better survival in pelvic fracture patients operated within 8 h. However, their number of patients was only 37 and the majority of them were operated with external fixation [16]. A surgical method with limited indications as a definitive fixation nowadays. Enninghorst et al. retrospectively analyzed selected pelvic fractures suitable for minimally invasive internal fixation (SI-screw and symphysis plate only) and used 24 h as a cut-off when comparing early and late surgery [18]. The early group (n = 18) was actually taken very early to surgery (mean 5.5 h), compared to the late group (n = 27) who had to wait 5 days (mean). They reported a trend for shorter hospital stay and decreased 24-hour red blood cell transfusion rate in the early group. Although their results seem to favor early surgery for this highly selected group of pelvic fracture patients, with such a limited number of patients in combination with the pronounced difference in time to surgery one must be careful when interpreting their conclusion. Vallier et al. retrospectively analyzed 645 patients with surgically treated pelvic or acetabular fractures comparing definitive fixation within, or after 24 h. Although a bit skewed (almost twice as many patients in the late group), their cohort was very similar to ours and their focus was on adverse events [12]. Some sub-analyses were done using 72 h as cut-off. They performed detailed analyses including the influence of concomitant injuries and injury severity on a number of adverse events, and in summary found significantly less pulmonary complications in the early surgery group, but no other differences in other adverse events. In addition, they reported shorter stay in intensive care unit for early surgery patients but no difference in total length of stay. Unfortunately, they did not report on reoperations, overall mortality or follow-up time. However, they concluded, as well as us, that early surgery of these patients is safe. This is also in line with another study from Vallier et al. on poly-trauma patients where they concluded that with a proper protocol for Early Appropriate Care (EAC), early fixation (within 36 h) of major orthopaedic injuries (femur, pelvic, acetabular, spine fractures) was associated with fewer complications and shorter hospital length of stay [22].

In a recent (2020) study by Parry et al., 130 patients with isolated acetabular fractures were analyzed to compare surgery before or after 48 h after admission [14]. Their focus was bleeding-related outcomes and among other findings they reported no significant differences in estimated blood loss. Although in contrast to our results (larger estimated blood loss in the late surgery group), their cohort consisted only of patients operated on with one surgical approach (no patient underwent double approaches at the same operation or day). We can only speculate about the reasons for our finding of lower estimated blood loss in the late surgery group as being a result from blood clotting over time, longer resuscitation and/or better surgical preoperative planning. However, although a significant difference, the absolute difference between the groups in median estimated blood loss was only 145 mL. As a secondary finding, we noted that the hemoglobin level at arrival was slightly but significantly higher in the early surgery group, which in theory could be beneficial for these patients making them less affected by subsequent intraoperative bleeding. Also, Dailey and Archdeacon reported no significant differences in estimated blood loss when comparing early (within 48 h) and late (> 48 h) surgery in 288 patients with acetabular fractures [11]. Their cohort also included only patients operated on using a single approach. Although we excluded patients who had simultaneous other major operations in the bleeding analyses, we think that our results are more generalizable in clinical practice as our cohort included an unselected mix of patients with both pelvic and acetabular fractures regardless of the number of surgical approaches that were used. In addition, we think that the unfavorable outcome with a small increase in bleeding in the early surgery group is of limited clinical importance.

Other authors have also used 48 h as a cut-off for patients with acetabular fractures referring to current recommendations regarding hip fracture surgery in geriatric patients [23, 24]. However, we do not think that experiences from surgery of hip fracture patients necessary can be applied on patients with pelvic or acetabular fractures in general. We did choose 72 h as our cut-off for this study as we think that it is a reasonable and achievable goal in our setting, as well as in many other. As the surgery of pelvic and acetabular fractures require complex multidisciplinary care in trauma centers, and only should be done by dedicated well-trained surgeons it can be hard to implement all days of the week, all year around. As an example, if a patient arrives on a Friday afternoon, the surgery must start at the latest on the following Sunday morning to be performed within 48 h. Furthermore, it is not only about availability of skilled surgeons, several factors such as adequate operating facilities, availability of skilled anesthesiologists and not least resources for resuscitation will all be of importance. The same cut-off (72 h) has been used by other authors [7, 12, 25]. In accordance with our results, Goldstein et al. reported that surgery within 72 h was safe in their series of patients with disrupted pelvic rings, although only including 33 patients [25]. Valier et al. reported that 55% of the patients operated > 72 h after injury who developed pneumonia actually had no underlying chest injury [12].

We found significant differences in age and proportion of high-energy trauma between the groups, with patients in the early group being slightly younger (mean 50 versus 56 years) and with 84% high-energy trauma in the early compared to 68% in the late surgery groups. This most likely reflects that younger patients are more likely to expose themselves to “high-energy activities” and was not correlated to an increased risk for reoperation or other adverse event in the regression analyzes.

There was no difference in the reoperation rates between the groups (23% in both groups), with a deep infection being the most common reason in both groups. Due to the small number of each type of reoperation in each group, no obvious pattern could be identified and further sub-analyses of this topic is hard to make within the context of this study. But we notice that although reoperation must be considered a major complication, none of the above mentioned studies have actually analyzed this as a part of their aims. We can just conclude that although 23% must be considered a high, or even unacceptable, number, it is quite within the range of other studies more focused on this topic [2, 26–29]. One must though bear in mind that there is a large variety in these studies regarding the follow-up time and also the definition of a reoperation.

We found a larger number of surgically treated acetabular (n = 228) compared to pelvic (n = 191) fractures. This ratio most probably differs between different institutions but corresponds with the findings in a recent national study including all pelvic and acetabular fractures in Sweden during 2010–2019 reporting an overall higher number of surgically treated acetabular (n = 1813) compared to pelvic (n = 1613) fractures [30].

Finally, in an interesting longitudinal study by Devaney et al., they report that the mean time to definitive fixation of pelvic and acetabular fracture patients at their institution has decreased from 116 h in the year 2009 to 54 h in the year 2018 [13]. They further reported that they saw no significant changes in mortality, length of hospital or intensive care unit stay for the overall cohort over the years.

### Strengths and limitations

A major strength of this study was the large number of included patients. Another strength was the relatively long follow-up time, allowing for the capture of late as well as early complications. All reviewing of medical charts and fracture classification was performed by the two authors, assuring consistency in collecting the data. There were several limitations with the study, whereof the main limitation was the retrospective design. This means that we cannot completely guarantee that some patients might have sustained a reoperation or adverse event at another hospital, although care was taken to include these when information was present. Unfortunately, we did not have data on ISS (injury severity score), surgical time or postoperative quality of fracture reduction. However, as ISS reflects the summary of several (up to three) injuries in different organ systems it does not necessarily influence the outcome of specific surgical procedures. Regarding surgery time, this can be difficult or even misleading to use in analyses as pelvic and acetabular surgery sometimes include dual patient positions (prone, supine and/or lateral) with time included for repositioning of the patient. Also, in poly-trauma cases when multiple surgical procedures/fracture operations are performed during the same session, the time used specifically for the pelvic/acetabular surgery can be difficult to estimate. So, although these variables could have been interesting to add in the analyses, we think that other used variables compensated for this. Finally, the single-center design of the study might limit the generalizability of the results.

## Conclusions

In summary, we think that with the results from our large study together with previous studies there now is convincing data to support early definitive fixation of pelvic and acetabular fractures. One can still debate what the exact cut-off (24, 48, 72 h etc.) should be, but, in a clinical setting this number is not important for the individual patient. The most important is rather an overall acceptance that these patients should be prioritized, and focus should be on quick resuscitation preoperatively.

## Data Availability

The datasets used and/or analysed during the current study are available from the corresponding author on reasonable request.

## List of abbreviations

APC: Anteroposterior compression
ASA: American society of anesthesiologists
CT: Computer tomography
GCS: Glasgow coma scale
LC: Lateral compression
SD: Standard deviation
IQR: Interquartile range
ISS: Injury severity score
OR: Odds ratio
THA: Total hip arthroplasty
VS: Vertical shear
